# Notification that new names of prokaryotes, new combinations, and new taxonomic opinions have appeared in volume 73, part 11 of the IJSEM

**DOI:** 10.1099/ijsem.0.006213

**Published:** 2024-03-04

**Authors:** Aharon Oren, Markus Göker

**Affiliations:** 1 The Institute of Life Sciences, The Hebrew University of Jerusalem, The Edmond J. Safra Campus, 9190401 Jerusalem, Israel; 2 Leibniz Institute DSMZ – German Collection of Microorganisms and Cell Cultures, Inhoffenstrasse 7B, 38124 Braunschweig, Germany

This listing of names of prokaryotes published in a previous issue of the *International Journal of Systematic and Evolutionary* (IJSEM) is provided as a service to microbiology to assist in the recognition of new names and new combinations. This procedure was proposed by the Judicial Commission [1]. The names given herein are listed according to the Rules of priority (i.e., time and date of publication of the articles on the journal’s platform and the order of valid publication of names in the original articles) [2].

**Table:** 

Order of article publication	Name/authors	Proposed as	Article no.	Page no.
1	*Aerococcus agrisoli* Sun *et al*. 2023	sp. nov.	006069	5
2	*Paenibacillus spongiae* Zhang *et al*. 2023	sp. nov.	006122	8
3	*Cutibacterium equinum* Yun *et al*. 2023	sp. nov.	006099	7
4	*Marixanthotalea* Fu *et al*. 2023	gen. nov.	006107	7
4	*Marixanthotalea marina* Fu *et al*. 2023	sp. nov.	006107	7
5	*Solibacillus daqui* Liang *et al*. 2023	sp. nov.	006109	5
6	*Bacillus mexicanus* de los Santos Villalobos *et al*. 2023	sp. nov.	006110	8
7	*Lacibacter sediminis* Zhuo *et al*. 2023	sp. nov.	006105	7
8	*Microbacterium festucae* Li *et al*. 2023	sp. nov.	006121	7
8	*Microbacterium nymphoidis* Li *et al*. 2023	sp. nov.	006121	8
9	*Salipaludibacillus daqingensis* Guo *et al*. 2023	sp. nov.	006126	6
10	*Kitasatospora fiedleri* Zimmermann *et al*. 2023	sp. nov.	006137	7
11	*Aeromicrobium duanguangcaii* Ye *et al*. 2023	sp. nov.	006118	6
11	*Aeromicrobium wangtongii* Ye *et al*. 2023	sp. nov.	006118	8
11	*Aeromicrobium senzhongii* Ye *et al*. 2023	sp. nov.	006118	8
12	*Jiella pelagia* Shin *et al*. 2023	sp. nov.	006139	5
13	*Pontibacter harenae* Osman *et al*. 2023	sp. nov.	006128	5
14	*Marinicella marina* Zhang *et al*. 2023	sp. nov.	006130	10
14	*Marinicella gelatinilytica* Zhang *et al*. 2023	sp. nov.	006130	10
15	*Sulfurovum mangrovi* Li *et al*. 2023	sp. nov.	006142	7
16	*Trinickia violacea* Gao *et al*. 2023	sp. nov.	006147	8
16	*Trinickia terrae* Gao *et al*. 2023	sp. nov.	006147	9
17	*Roseateles amylovorans* Guliayeva *et al*. 2023	sp. nov.	006133	6
18	*Lentilactobacillus dabitei* (Li *et al*. 2022) Wang and Gu 2023	comb. nov., elevation in rank of *Lentilactobacillus rapi* subsp. *dabitei* Li *et al*. 2022	006149	5
19	*Flectobacillus longus* Lu *et al*. 2023	sp. nov.	006148	5
19	*Flectobacillus rivi* Lu *et al*. 2023	sp. nov.	006148	7
20	*Salinigranum marinum* Cheng *et al*. 2023	sp. nov.	006143	6
20	*Halohasta salina* Cheng *et al*. 2023	sp. nov.	006143	7
21	*Nocardioides cremeus* Wang *et al*. 2023	sp. nov.	006138	5
21	*Nocardioides abyssi* Wang *et al*. 2023	sp. nov.	006138	6
21	*Nocardioides oceani* Wang *et al*. 2023	sp. nov.	006138	7
22	*Pseudalkalibacillus spartinae* Liu *et al*. 2023	sp. nov.	006159	5
22	*Pseudalkalibacillus sedimenti* Liu *et al*. 2023	sp. nov.	006159	6
23	*Alteriqipengyuania flavescens* Zhang *et al*. 2023	sp. nov.	006161	7
24	*Gardnerella pickettii* Sousa *et al*. 2023	sp. nov.	006140	8
24	*Gardnerella greenwoodii* Sousa *et al*. 2023	sp. nov.	006140	8
25	*Hydrogenimonas cancrithermarum* Mino *et al*. 2023	sp. nov.	006132	9
26	*Tepidibacter hydrothermalis* Dai *et al*. 2023	sp. nov.	006151	7
27	*Candidatus* Kirkpatrickella Henry *et al*. 2023∗	gen nov.	006111	8
27	*Candidatus* Kirkpatrickella diaphorinae Henry *et al*. 2023∗	sp. nov.	006111	8
28	*Microcella humidisoli* Molina Ayala and Kim 2023	sp. nov.	006150	6
28	*Microcella daejeonensis* Molina Ayala and Kim 2023	sp. nov.	006150	7
28	*Microcella pacifica* (Qin *et al*. 2021) Molina Ayala and Kim 2023	comb. nov. [basonym: *Marinisubtilis pacificus* Qin *et al*. 2021]	006150	8
29	*Tumebacillus lacus* Zhang *et al*. 2023	sp. nov.	006153	7
30	*Pinibacter soli* Huq *et al*. 2023	sp. nov.	006136	7
31	*Curtobacterium caseinilyticum* Feng *et al*. 2023	sp. nov.	006152	5
31	*Curtobacterium citri* Feng *et al*. 2023	sp. nov.	006152	6
31	*Curtobacterium subtropicum* Feng *et al*. 2023	sp. nov.	006152	6
32	*Methanovulcanius* Chien *et al*. 2023	gen. nov.	006164	6
32	*Methanovulcanius yangii* Chien *et al*. 2023	sp. nov.	006164	7
33	*Robiginitalea aurantiaca* Zhou *et al*. 2023	sp. nov.	006155	8
33	*Algoriphagus sediminis* Zhou *et al*. 2023	sp. nov.	006155	9
34	*Lysinibacillus irui* Akintayo *et al*. 2023	sp. nov.	006167	8
35	*Ligilactobacillus cholophilus* Ren *et al*. 2023	sp. nov.	006160	8
36	*Neopusillimonas aromaticivorans* Lin *et al*. 2023	sp. nov.	006146	7
37	*Thermobacterium* Chen *et al*. 2023 non *Thermobacterium* Buchanan 1918	gen. nov.	006166	5
37	*Thermobacterium salinum* Chen *et al*. 2023	sp. nov.	006166	5
38	*Mycoplasma phocimorsus* Skafte-Holm *et al*. 2023	sp. nov.	006163	8
39	*Robiginitalea aestuariiviva* Cao *et al*. 2023	sp. nov.	006170	7
40	*Shimia litoralis* (Kim et al. 2008) Zhang *et al*. 2023	comb. nov. [basonym: *Pelagicola litoralis* Kim *et al*. 2008]†	006156	9
40	*Aliiroseovarius lamellibrachiae* (Nogi *et al*. 2017) Zhang *et al*. 2023	comb. nov. [basonym: *Planktotalea lamellibrachiae* Nogi *et al*. 2017]	006156	10
40	*Maritimibacter fusiformis* (Wang *et al*. 2021) Zhang *et al*. 2023	comb. nov. [basonym: *Muriiphilus fusiformis* Wang *et al*. 2021]	006156	10
40	*Palleronia caenipelagi* (Park *et al*. 2019) Zhang *et al*. 2023	comb. nov. [basonym: *Paenimaribius caenipelagi* Park *et al*. 2019]	006156	10
40	*Thalassobius vesicularis* (Hameed *et al*. 2014) Zhang *et al*. 2023	comb. nov. [basonym: *Youngimonas vesicularis* Hameed *et al*. 2014]	006156	10
40	*Pseudooceanicola sediminis* (Zhang *et al*. 2019) Zhang *et al*. 2023	comb. nov. [basonym: *Pseudopuniceibacterium sediminis* Zhang *et al*. 2019]	006156	10
40	*Pseudooceanicola antarcticus* (Liao *et al*. 2021) Zhang *et al*. 2023	comb. nov. [basonym: *Pseudopuniceibacterium antarcticum* Liao *et al*. 2021]	006156	10
40	*Roseovarius dicentrarchi* (Li *et al*. 2020) Zhang *et al*. 2023	comb. nov. [basonym: *Pelagivirga dicentrarchi* Li *et al*. 2020]	006156	11
41	*Blautia parvula* Miura *et al*. 2023	sp. nov.	005871	5
42	*Methanolobus mangrovi* Zhou *et al*. 2023	sp. nov.	006169	6
42	*Methanolobus sediminis* Zhou *et al*. 2023	sp. nov.	006169	6
43	*Muricauda myxillae* Moon *et al*. 2023	sp. nov.‡	006040	7
43	*Muricauda symbiotica* Moon *et al*. 2023	nom. nov. [basonym: *Flagellimonas hymeniacidonis* Shin and Park 2022]‡	006040	8
44	*Marinihelvus* Zhang *et al*. 2023	gen. nov.	006165	8
44	*Marinihelvus fidelis* Zhang *et al*. 2023	sp. nov.	006165	9
45	*Paenibacillus caseinilyticus* Lee *et al*. 2023	sp. nov.	006171	6
46	*Gordonia metallireducens* Grimm *et al*. 2023	sp. nov.	006176	8
47	*Tessaracoccus caeni* Wang *et al*. 2023	sp. nov.	006113	6
48	*Haloarcula terrestris* Straková *et al*. 2023	sp. nov.	006157	9
49	*Mangrovimonas aestuarii* Zhang *et al*. 2023	sp. nov.	006120	6
50	*Shiella* Liu *et al*. 2023	gen. nov.	006175	7
50	*Shiella aurantiaca* Liu *et al*. 2023	sp. nov.	006175	7
50	*Shiellaceae* Liu *et al*. 2023	fam. nov.	006175	8
51	*Vibrio chanodichtyis* Wu *et al*. 2023	sp. nov.	006117	4
52	*Actinoallomurus soli* Chantavorakit *et al*. 2023	sp. nov.	006177	7
52	*Actinoallomurus rhizosphaericola* Chantavorakit *et al*. 2023	sp. nov.	006177	7
53	*Blastococcus carthaginiensis* Kammoun *et al*. 2023	sp. nov.	006178	7
54	*Vibrio methylphosphonaticus* Wang *et al*. 2023	sp. nov.	006183	5
55	*Parabacteroides leei* Byun *et al*. 2023	sp. nov.	006187	4
56	*Psychrobacillus antarcticus* da Silva *et al*. 2023	sp. nov.	006181	4
57	*Helicobacter ibis* Lopez-Cantillo *et al*. 2023	sp. nov.	005983	7
58	*Marinitoga aeolica* Postec *et al*. 2023	sp. nov.	006186	7
59	*Paenibacillus polygoni* Long *et al*. 2023	sp. nov.	006185	6
60	*Empedobacter sedimenti* Ruan *et al*. 2023	sp. nov.	006182	4
61	*Arthrobacter zhaoxinii* Zhang *et al*. 2023	sp. nov.	006168	7
61	*Arthrobacter jinronghuae* Zhang *et al*. 2023	sp. nov.	006168	8

∗*Candidatus* names are not validly published.

†Illegitimate name; later homonym of *Pseudooceanicola antarcticus* (Huo *et al*. 2014) Lai *et al*. 2015.

‡The genus name *Muricauda* Bruns *et al*. 2001 is illegitimate.

The following minor corrections should be made in the Notification List for Volume 73, part 7 of the IJSEM [3]:

**Table:** 

Order of article publication	Name/authors	Proposed as	Article no.	Page no.
6	*Holtiella tumoricola* Allen-Vercou *et al*. 2023 should be corrected to *Holtiella tumoricola* Allen-Vercoe *et al*. 2023	gen. nov.	005958	5
6	*Holtiella tumoricola* Allen-Vercou *et al*. 2023 should be corrected to *Holtiella tumoricola* Allen-Vercoe *et al*. 2023	sp. nov.	005958	5
8	*Oikeobacillus* Rao *et al*. 2023 should be corrected to *Oikeobacillus* Narsing Rao *et al*. 2023	gen. nov.	005961	8
19	*Asticcacaulis aquaticus* Chen *et al*. 2023 should be corrected to *Asticcacaulis aquaticus* Lu *et al*. 2023	sp. nov.	005974	6
19	*Asticcacaulis currens* Chen *et al*. 2023 should be corrected to *Asticcacaulis currens* Lu *et al*. 2023	sp. nov.	005974	6
19	*Asticcacaulis machinosus* Chen *et al*. 2023 should be corrected to *Asticcacaulis machinosus* Lu *et al*. 2023	sp. nov.	005974	7

The following minor corrections should be made in the Notification List for Volume 73, part 9 of the IJSEM [4]:

**Table:** 

Order of article publication	Name/authors	Proposed as	Article no.	Page no.
15	*Allofranklinella schreckenbergeri* (Bernard *et al*. 2022)) Deshmukh and Oren 2023 should be corrected to *Allofranklinella schreckenbergeri* (Bernard *et al*. 2022) Deshmukh and Oren 2023	sp. nov.	006023	9

The following taxonomic opinions (i.e., the emendation of circumscriptions or the creation of synonyms) do not affect the status of a name under the International Code of Nomenclature of Prokaryotes. These taxonomic opinions can neither be considered as approved nor as disapproved by the International Committee on Systematics of Prokaryotes.

**Table:** 

Name/authors:	Article no.	Page no.
*Deinococcus saudiensis* Hussain *et al*. 2016 pro synon. *Deinococcus soli* Cha *et al*. 2014	006162	4
*Flectobacillus rhizosphaerae* Ramaprasad *et al*. 2015 pro synon. *Flectobacillus roseus* Sheu *et al*. 2009	006148	5
*Haloarcula argentinensis* Ihara *et al*. 1997 emend. Straková *et al*. 2023	006157	9
*Hydrogenimonas* Takai *et al*. 2004 emend. Mino *et al*. 2023	006132	9
*Microcella* Tiago *et al*. 2005 emend. Molina Ayala and Kim 2023	006150	8
